# The prognostic value of circRNAs for gastric cancer: A systematic review and meta‐analysis

**DOI:** 10.1002/cam4.3497

**Published:** 2020-10-27

**Authors:** Hui Chen, Chengtong Liang, Xuechun Wang, Yu Liu, Zhanjun Yang, Ming Shen, Chongxu Han, Chuanli Ren

**Affiliations:** ^1^ Geriatric Medicine Northern Jiangsu People’s Hospital Yangzhou China; ^2^ Department of Laboratory Medicine Clinical Medical College of Yangzhou University Yangzhou China; ^3^ Department of Laboratory Medicine Dalian Medical University Dalian China; ^4^ Department of Laboratory Medicine Medical College of Yangzhou University Yangzhou China; ^5^ School of Chemistry and Chemical Engineering Yangzhou University Yangzhou China

**Keywords:** circRNAs, gastric cancer, meta‐analysis, prognosis

## Abstract

Gastric cancer is the third leading cause of cancer‐related deaths worldwide. Novel biomarkers circRNAs can play an important role in the development of gastric cancer as oncogenes or tumor suppressor genes. The purpose of this study was to clarify the relationship between the abnormal expression of multiple circRNAs and their prognostic value in gastric cancer patients through a meta‐analysis. We researched articles reporting the relationship between circRNAs and the prognosis of gastric cancer published in PubMed, Cochrane, Embase, Web of Science, Wanfang, CNKI, and VIP databases before 31 December 2019. Thirty‐five articles were selected for the meta‐analysis, involving 3135 gastric cancer patients. The total HR values (95% CI) of OS and DFS related to highly expressed circRNAs that indicated worse prognosis were 1.83 (1.64‐2.03; *p* < 0.001) and 1.66 (1.33‐2.07; *p* < 0.001), respectively. The total HR (95% CI) of OS and DFS related to highly expressed circRNAs that indicated better prognosis was 0.54 (0.45‐0.66; *p* < 0.001) and 0.58 (0.43‐0.78; *p* < 0.001), respectively. Two panels of five circRNAs predicted a more considerable HR value (circ_0009910, hsa_circ_0000467, hsa_circ_0065149, hsa_circ_0081143, and circDLST; and circSMARCA5, circLMTK2, hsa_circ_0001017, hsa_circ_0061276, and circ‐KIAA1244). The results of the meta‐analysis were 2.63 (2.08‐3.33; *p* < 0.001) and 0.39 (0.27‐0.59; *p* < 0.001) for OS, respectively. The two panels of dysregulated circRNAs can be considered as more suitable potential prognostic tumor biomarkers in patients with gastric cancer because of their larger HR values.

## Graphical abstract

We obtained the HR value which combined analysis of survival outcomes and time from gastric cancer patients in each clinical trial as a reference to judge the prognostic value of circRNAs in gastric cancer. We found that two panels of five circRNAs showed a greater prognostic value: 2.63(2.08‐3.33), *p* < 0.001 (circ_00099 10, hsa_circ_0000467, hsa_circ_0065149, hsa_circ_0081143, circDLST); 0.39(0.27‐ 0.59), *p* < 0.001 (circSMARCA5, circLMTK2, hsa_circ_0001017, hsa_circ_0061276, circ‐KIAA1244).

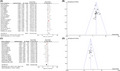

## 1 INTRODUCTION

Gastric cancer (GC) is one of the most common cancers globally. In 2018, there were more than 1 million new cases, and the estimated number of deaths reached 783,000 (equivalent to 1 in 12 deaths worldwide). GC is the fifth most common type of cancer worldwide. It is also the third leading cause of cancer‐related deaths.^1^ Approximately 70% of new cases are concentrated in developing countries, especially China. According to these studies, China has a high incidence of GC. The incidence of GC in men is second to lung cancer, while the probability of women being diagnosed with GC is only lower than that of breast cancer and lung cancer, ranking third in the incidence of malignant tumors over the same period.^2^

The occurrence and development of GC are the results of multiple factors. It has been reported that *Helicobacter pylori* infection and a family history of GC are the main risk factors.^3^ In addition, another study stated that poor eating habits (high‐salt diet, preserved food, fried food), unhealthy lifestyle (alcoholism, cigarettes, excessive overeating), history of stomach surgery, and occupational exposure history (cement, mineral dust, chromium) can promote the development of GC.^4^, ^5^ However, advanced GC can be caused by extensive infiltration of tumor tissue in multiple organs, leading to a poor prognosis. Identifying accurate, convenient, and economical biomarkers to determine prognosis has always been a hotspot in cancer research. Liquid biomarkers have advantages such as noninvasiveness, painlessness, convenient material acquisition, and continuous testing in vitro. They are not only widely used in clinical applications, but also continue to be extensively researched. In recent years, the study of liquid biomarkers has expanded from the traditional protein level to the molecular level. However, because of the limitations of biological characteristics and experimental conditions, many biomarkers have the characteristics of low experimental sensitivity and low repeatability of detection. Therefore, biomarkers with high sensitivity, excellent specificity, and high repeatability that accurately reflect patient prognosis and have the ability to guide treatment require further investigation.

CircRNAs are a recently identified type of RNA that have a covalent circular structure. With the emergence of high‐throughput sequencing, exoenzyme‐based enrichment tools, and bioinformatics, the detection amount and types of circRNAs have increased rapidly, and a large number of circRNA families have been found in various animals, plants, and humans. Memczak and Hansen have made breakthrough progress in the study of the biological function of circRNAs, and proposed that circRNAs can function as miRNA sponges in gene expression, gene regulation, and posttranscriptional processes for the first time.^6^, ^7^ Many studies have since shown that circRNAs have an important role in the development of tumors. CircRNAs can affect gene expression and transcription in many diseases including GC through various mechanisms.^8^ Biological experiments revealed that many circRNAs contain miRNA binding sites, which can be used as miRNA sponges to inhibit or promote the progression of GC. In addition, circRNAs regulate the transcription of parental genes, participating in protein interaction and translating proteins.^9^ A variety of circRNAs can function as oncogenes or tumor suppressor genes. As circRNAs are characterized by their high abundance, high stability, conservatism, and tissue specificity, they are considered to have excellent potential value, especially as biomarkers for diagnosis and prognosis. Treatment targets are also the focus of research.^8^, ^10^ The dysregulation of specific circRNAs as molecular biomarkers is closely related to the prognosis of GC patients.

However, because single‐center, small‐sample studies with different experimental methods and standards were adopted by different research centers, the ability to evaluate the relationship between the expression of multiple circRNAs and the prognosis of GC patients is limited. To the best of our knowledge, this is the largest study to further elucidate the relationship between the expression of multiple circRNAs and the prognosis of GC, so as to advance our understanding of the prognostic value of circRNAs and promote the development of targeted therapy with circRNAs.

## 2 METHODS

### 2.1 Search strategy

We performed searches for studies on the relationship between circRNAs and the prognosis of GC in PubMed, Cochrane, EMBASE, Web of Science, Wanfang, China National Knowledge Infrastructure (CNKI), and China Science and Technology Journal (VIP) databases published before 31 December 2019. The specific retrieval strategies were as follows: #1 “RNA, circular” OR “circular RNA*” OR “circRNA*”; #2 “stomach neoplasms” OR “gastric cancer” OR “stomach cancers” OR “gastric carcinoma” OR “advanced gastric cancer”; and #3 “prognosis” OR “prediction” OR “survival” OR “hazard ratio” OR “mortality” OR “follow up studies.” The search formula was as follows: #1 AND #2 AND #3 (combination of subject words and free words).

### 2.2 Inclusion and exclusion criteria:

The inclusion criteria were as follows: (a) patients with GC diagnosed pathologically; (b) prognosis of patients with stage I‐IV disease was predicted based on the expression level of circRNAs; (c) expression level of circRNAs can be divided into high expression and low expression; and (d) we could directly obtain the overall survival (OS), disease‐free survival (DFS), hazard ratio (HR), and 95% confidence interval (95% CI) values from the text or extract them from the Kaplan‐Meier curve. The exclusion criteria were as follows: (a) reviews, letters, case reports, and nonclinical related studies; (b) non‐English, non‐Chinese, nonhuman studies, and articles without data; and (c) duplicate articles.

## 3 DATA EXTRACTION

According to the above inclusion and exclusion criteria, two researchers independently selected articles from a large number of literature and extracted and entered relevant data from the articles that met the criteria. In cases of disagreement, the final results were determined by discussion between the two researchers, and the results were recorded after reaching a consensus. Articles that were clearly unrelated to the research theme were excluded by reading the title/abstract, and then, the full text of the remaining articles was read after excluding such articles. The literature was classified according to inclusion and exclusion criteria and determined as to whether it was eventually included. The content of the data extraction mainly included basic information of the included studies (including first author, type of circRNAs, publication time, country, number of patients, test methods, sample types, cutoff value) and survival data (including follow‐up time, HR, and 95% CI). We used the NOS (Newcastle‐Ottawa Scale) scale for quality evaluation and assigned the articles from 0 to 9 according to the content of the literature and judgment criteria.

### 3.1 Statistical analysis

Survival analysis is an analysis method that takes the patient's death as the end point of observation and combines the survival time. We mainly obtained the HR value from the survival analysis curve. Our methods of obtaining HR were as follows: (a) If the HR and 95% CI had been given in the literature, it could be collected directly. (b) If the Kaplan‐Meier survival curve was given in the literature, we used Engauge digitizer 4.1 software to read the survival data from the survival curve, and then, calculated the HR and 95% credibility interval using the method described by Tierney.^11^

We used the *I* ^2^ statistic method to test the heterogeneity between the included studies. If the heterogeneity test results were *p* > 0.1 and *I* ^2^ < 50%, the included studies were considered as having no obvious heterogeneity, and the fixed effects model (FEM) was used to combine the HR value. If *p* ≤ 0.1 and *I^2^ *> 50%, it was considered that there was obvious heterogeneity between the included studies, and a random effects model (REM) was used to merge the HR value. At this time, the source of heterogeneity needed to be further explored and a subgroup analysis was performed to determine whether the heterogeneity could be explained by clinical heterogeneity or methodological heterogeneity. It was also feasible to use a sensitivity analysis to identify the impact of a single research result on the meta‐analysis conclusion. If the heterogeneity was too large or the original data could not be obtained, only a descriptive analysis was performed instead of a meta‐analysis. RevMan 5.3 software was used to combine the effect size and calculate *I* ^2^ statistics to judge the heterogeneity.

## 4 RESULTS

### 4.1 Search results

**FIGURE 1:**
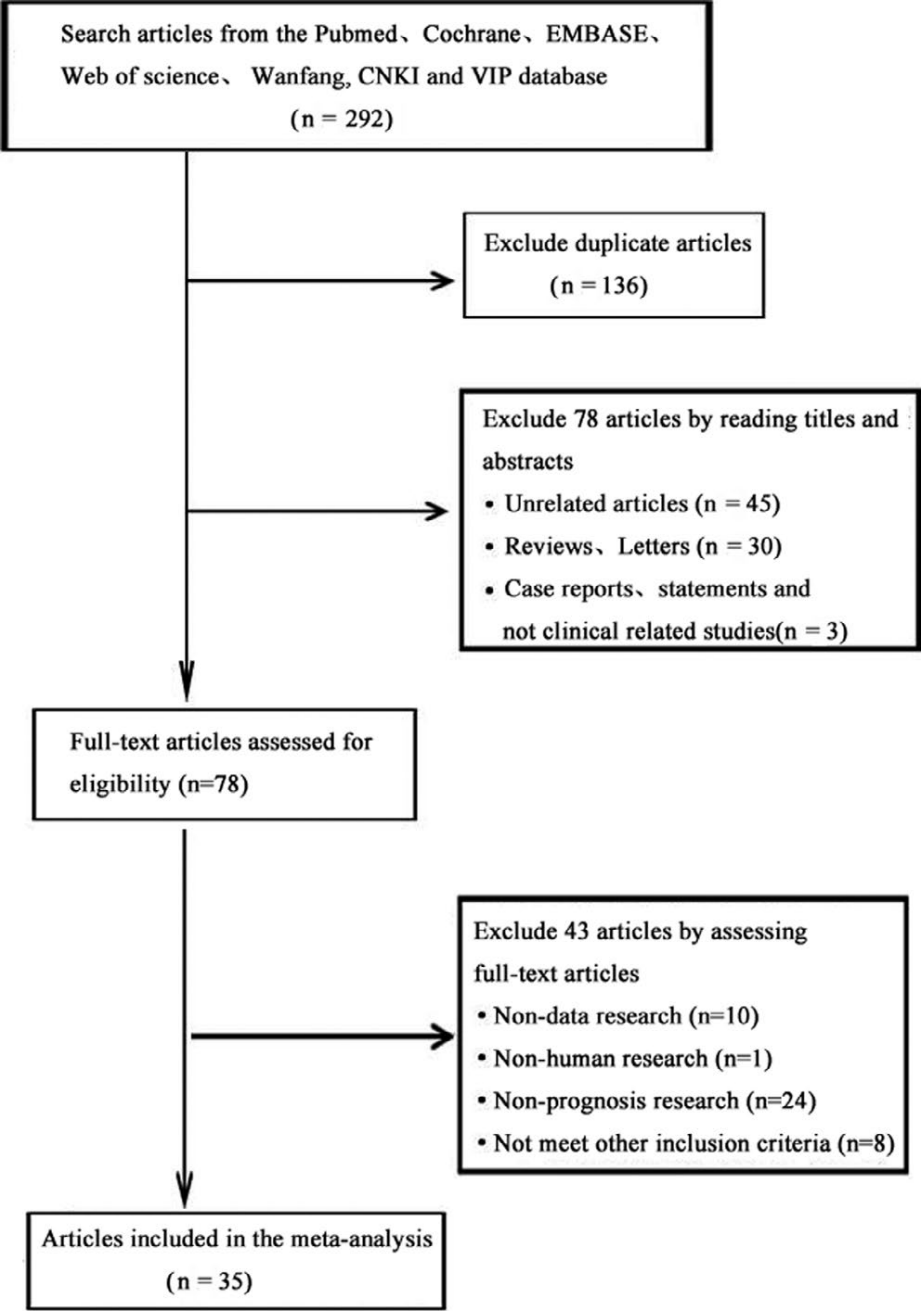
Flow chart of the study selection process

After searching for articles according to the specific detection strategy, 292 articles were initially screened. One hundred and thirty‐six duplicate articles were deleted after importing into Endnote Document Manager. Seventy‐eight articles including irrelevant articles, reviews, letters, case reports, and nonclinical related studies were excluded after carefully reading the titles and abstracts. Forty‐three articles including non‐data studies, non‐prognosis studies, nonhuman studies, and noncompliant studies were excluded through intensive reading of the full text. Finally, 35 literature studies with a total of 3135 patients were included in this meta‐analysis.^8^, ^10^, ^12^, ^13^, ^14^, ^15^, ^16^, ^17^, ^18^, ^19^, ^20^, ^21^, ^22^, ^23^, ^24^, ^25^, ^26^, ^27^, ^28^, ^29^, ^30^, ^31^, ^32^, ^33^, ^34^, ^35^, ^36^, ^37^, ^38^, ^39^, ^40^, ^41^, ^42^, ^43^, ^44^ The process is shown in Figure 1.

### 4.2 Characteristics of included studies

The meta‐analysis of 35 articles, involving 3135 people, was the largest sample study for predicting the effect of circRNAs on the prognosis of GC to date. All included studies were from China and were published between 2016 and 2019. HRs and 95% CIs were provided in 13 articles and survival curves were provided in 22 articles. The expression levels of circRNAs included in all studies were detected using qRT‐PCR technology. The literature quality evaluation was independently completed by two researchers by referring to the relevant literature. Cutoff values of high or low circRNA expression were mostly median or mean. The average score of the literature was 7.9. The highest score was 9 and the lowest was 7. The details of these characteristics are shown in Table 1.

**TABLE 1:** Main characteristics of circRNAs studies for prognosis analysis of gastric cancer

References	circRNAs (n = 40)	Year	Nation	Number (n = 3135)	OS		Cutoff value	Sample types	Follow‐up NOS (month)	
					HR	95% CI				
Chen et al.	circPVT1^a^	2016	China	187	0.59	0.41‐0.86	2‐fold	Tissue	100	8
Li et al.	hsa_circ_0001017^b^	2017	China	278	0.41	0.16‐0.94	median	Plasma	60	7
Li et al.	hsa_circ_0061276^b^	2017	China	278	0.43	0.18‐0.93	median	Plasma	60	7
Pan et al.	ciRS−7^a^	2017	China	102	2.03	1.20‐4.11	median	Tissue	60	8
Zhang et al.	circRNA_100269^b^	2017	China	112	0.74	0.34‐1.58	NA	Tissue	50	7
Zhang et al.	Circular RNA_LARP4	2017	China	80	0.53	0.24‐0.99	2‐fold	Tissue	150	8
Li et al.	circ_0056618^a^	2018	China	32	1.67	0.61‐3.56	NA	Tissue	100	8
Li et al	circRBMS3^a^	2018	China	69	1.95	1.06‐3.60	2‐fold	Tissue	60	8
Liu et al.	circ_0009910 ^a^	2018	China	129	2.35	1.67‐3.77	NA	Tissue	60	7
Liu et al.	circular RNA YAP1^b^	2018	China	80	0.64	0.27‐1.53	13.37^e^	Tissue	120	8
Lu et al.	hsa_circ_0000467^a^	2018	China	51	2.68	1.42‐4.79	median	Tissue	50	7
Ouyang et al.	circPDSS1^a^	2018	China	20	1.65	0.50‐3.49	2‐fold	Tissue	60	8
Sun et al.	circPVRL3^b^	2018	China	62	0.44	0.20‐0.96	NA	Tissue	120	8
Tang et al.	circ‐KIAA1244^b^	2018	China	87	0.41	0.08‐0.92	1.443^e^	Plasma	40	8
Cai et al.	circSMARCA5^b^	2019	China	60	0.38	0.15‐0.95	median	Tissue	40	8
Cai et al.	circHECTD1^a^	2019	China	50	1.58	0.72‐2.80	2‐fold	Tissue	50	8
Du et al.	circ‐PRMT5^a^	2019	China	90	2.26	1.14‐4.47	median	Tissue	167	8
Ding et al.	circ‐DONSON^a^	2019	China	142	1.80	1.01‐3.61	NA	Tissue	120	8
He et al.	circLMTK2^b^	2019	China	111	0.36	0.17‐0.76	median	Tissue	100	8
Li et al.	circ‐ERBB2^a^	2019	China	58	1.57	0.57‐4.29	median	Tissue	60	8
Liu et al.	circHIPK3^a^	2019	China	53	1.78	1.12‐3.22	NA	Tissue	80	8
Lu et al.	circ‐RanGAP1^a^		China	97	1.43	1.05‐2.27	2‐fold	Tissue	60	8
Rong et al.	circPSMC3^b^	2019	China	106	0.59	0.30‐0.95	2‐fold	Tissue	50	8
Shao et al.	Hsa_circ_0065149^a^	2019	China	96	3.15	1.23‐5.04	10.19^e^	Tissue	72	8
Tao et al.	hsa_circ_0000419^b^	2019	China	96	0.81	0.18‐1.57	8.14^e^	Tissue	60	7
Wang et al.	circLMTK2^a^	2019	China	121	1.75	1.02‐3.13	median	Tissue	100	9
Wang et al.	circOSBPL10^a^	2019	China	70	1.61	0.79‐3.29	NA	Tissue	70	8
Wang et al.	Five‐circRNAs^a^, ^c^	2019	China	192	1.63	1.36‐1.95	median	Tissue	160	9
Wu et al.	circ‐PRKCI^a^	2019	China	50	1.64	0.57‐2.83	NA	Tissue	60	8
Wu et al.	circZNF609^a^	2019	China	80	1.58	0.78‐2.91	1.124^e^	Tissue	200	8
Wen et al.	Hsa_circ_0001614^b^	2019	China	63	0.53	0.22‐1.24	NA	Tissue	NA^d^	8
Wu et al.	circ‐DCAF6^a^	2019	China	62	2.06	1.06‐4.01	mean	Tissue	60	7
Xue et al.	hsa_circ_0081143^a^	2019	China	30	2.37	1.72‐ 4.20	2‐fold	Tissue	60	8
Yang et al.	hsa_circ_0005556^b^	2019	China	100	0.67	0.35‐1.28	NA	Tissue	72	8
Zhang et al.	circDLST^a^	2019	China	71	3.76	1.46‐5.67	NA	Tissue	100	8
Zhang et al.	Hsa_circ_0067997^a^	2019	China	48	1.47	0.51‐4.22	1.5‐fold	Tissue	50	8

All circRNAs was detected by qRT‐PCR.

^a^ Oncogene, Upregulated in gastric cancer tissue or plasma;

^b^ Suppressor, Downregulated in gastric cancer tissue or plasma;

^c^ hsa_circ_0103398, hsa_circ_0119099, hsa_circ_0121124, hsa_circ_0127859, and hsa_circ_0139915;

^d^ NA: Not application;

^e^ The cutoff value of ROC curve

### 4.3 Effect of the expression level of circRNAs on the overall survival of GC

**FIGURE 2:**
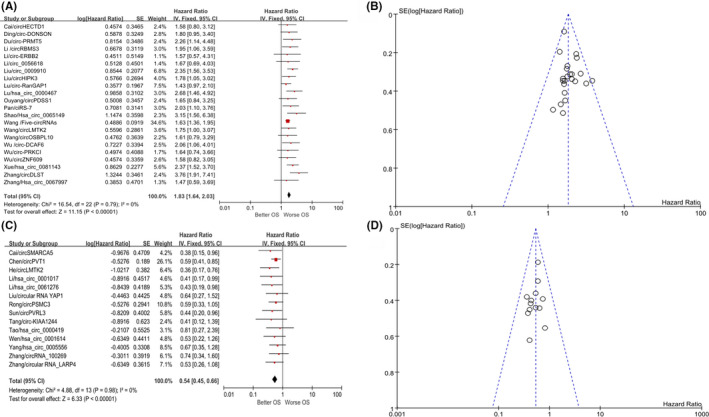
Forest and funnel plots for the prognostic value of circRNAs in overall survival (OS) of gastric cancer patients. High‐expressing circRNAs indicate worse prognosis (A, B) or better prognosis (C, D) for gastric cancer patients

**FIGURE 3:**
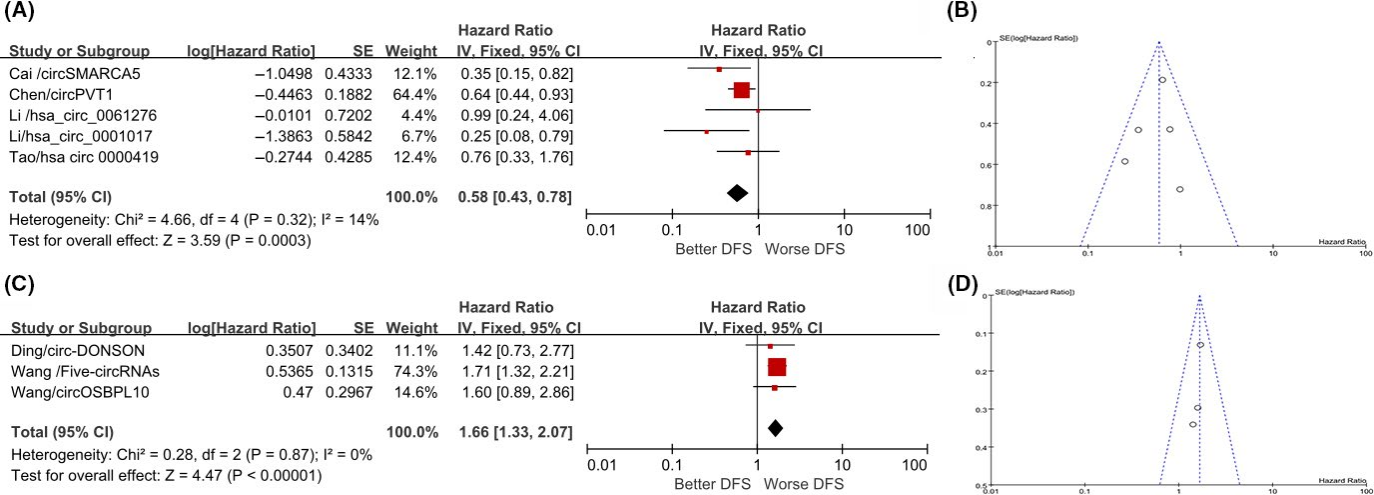
Forest and funnel plots of five circRNAs with maximum (A, B) and minimum (C, D) HR values for gastric cancer

After in‐depth reading of the included 35 articles, we found that 22 articles reported that circRNAs are highly expressed in GC tissues and are negatively correlated with patient prognosis. After quantitative analysis, high expression of these circRNAs indicated poor prognosis for GC patients (vs. low‐expression group: HR = 1.83; 95% CI, 1.64‐2.03; *p* < 0.001). *I* ^2^ = 0 indicated that the research results were highly reliable (Figure 2A); a funnel plot is shown in Figure 2B. At the same time, another 13 articles reported that circRNAs are lowly expressed in GC tissues and have a positive correlation with patient prognosis. The total HR value of the high‐expression group vs. the low‐expression group could be obtained (HR = 0.54; 95% CI, 0.45‐0.66; *p* < 0.001). There was also no heterogeneity, indicating that the research results were highly reliable (Figure 2C); a funnel plot is shown in Figure 2D. A panel of five circRNAs predicted a greater HR (circ_0009910, hsa_circ_0000467, hsa_circ_0065149, hsa_circ_0081143, and circDLST), which is displayed in Figure 3A. The following five circRNAs showed a smaller HR: circSMARCA5, circLMTK2, hsa_circ_0001017, hsa_circ_0061276, and circ‐KIAA1244 (Figure 3C). The results of the meta‐analysis were 2.63 (2.08‐3.33; *p* < 0.001) and 0.39 (0.27‐0.59; *p* < 0.001), respectively. Figure 3B,D show the respective funnel plots. In summary, circRNAs can be used as biological markers to judge the prognosis of GC patients. The roles of the included circRNAs in the development of GC and the specific mechanisms are shown in Table 2.

**TABLE 2:** The role of circRNAs in the development of gastric cancer

circRNAs (n = 40)	Gene symbol	Chromosome	Impact on functions of cells	Mechanism
circ‐PRMT5^a^	PRMT5	chr14	Proliferation/apoptosis	Sponging miR−145 and miR−1304 to upregulate MYC
circ‐PRKCI^a^	PRKCI	/	Proliferation/invasion	Binding to microRNA−545
circ‐DONSON^a^	DONSON	chr21q22.11	Proliferation/migration/ invasion/ apoptosis	NURF complex dependent activation of transcription factor SOX4
circHECTD1^a^	HECTD1	/	Glutaminolysis/proliferation/migration/ invasion	Targeting miR−1256 and activating β‐catenin/c‐Myc signaling to facilitates glutaminolysis
circZNF609^a^	ZNF609	/	Proliferation/ migration	Sponging miR−483‐3p and regulating CDK6
circ_0056618^a^	/	/	Proliferation/ metastasis	Regulating miR−206/CXCR4
circRBMS3^a^	RBMS3	chr3	Proliferation/invasion	Regulating miR−153‐SNAI1 axis
circDLST^a^	DLST	chr 14q24.3	Proliferation/Colony formation	Sponging miR−502‐5p and activating the NRAS/ MEK1/ ERK1/2 signaling
circOSBPL10^a^	OSBPL10	chr3	Proliferation/invasion/ migration	A miRNA sponge for miR−136‐5P
Five‐circRNAs^a^, ^c^	/	/	/	One carbon pool by folate pathway
hsa_circ_0000467^a^	SKA3	chr13	Proliferation/migration/invasion	Tumor promoter
circ‐ERBB2^a^	ERBB2	/	Proliferation/apoptosis, migration/invasion	MiR−503/CACUL1 and miR−637/MMP−19 signaling
ciRS−7^a^	/	/	Proliferation/metastasis/ colony formation	Abrogates the tumor suppressive effect of miR−7 via PTEN/PI3 K/AKT signaling pathway
Hsa_circ_0065149^a^	SETD2	chr3	/	Harbor hsa‐miR−197‐5p, hsa‐miR−222‐3p, hsa‐miR−330‐5p, and hsa‐miR−486‐3p seed sequences
circHIPK3^a^	HIPK3	/	Proliferate/migrate	Regulating the Wnt/β‐catenin pathway
Hsa_circ_0067997^a^	FNDC3B	/	Viability/colony formation/invasive ability	Inhibition of miR−515‐5p and activation of X chromosome‐linked inhibitor of apoptosis (XIAP)
circ_0009910^a^	/	/	Proliferation/migration/invasion	Decreased expression of E‐cadherin and increased expression of N‐cadherin and snail
circLMTK2^a^	LMTK2	chr7	Proliferation/metastasis	A sponge of miR−150‐5p
circ‐RanGAP1^a^	RANGAP1	chr22q13.2	Invasion/metastases	Regulates VEGFA expression by targeting miR−877‐3p
circ‐DCAF6^a^	/	chr1	Proliferation/apoptosis	Sponging miR−1231 and miR−1256
hsa_circ_0081143^a^	/	/	cisplatin resistance	Targeting miR−646/CDK6 pathway
circPDSS1^a^	PDSS1	/	apoptosis	Sponging miR−186‐5p and modulating NEK2
circPVT1^a^	PVT1	chr8q24	Proliferation	A sponge for members of the miR−125 family
circular RNA YAP1^b^	YAP1	/	Proliferation/invasion	Regulating the miR−367‐5p/p27^Kip1^axis
circPSMC3^b^	PSMC3	/	Proliferation/metastasis	Acting as a competitive endogenous RNA through sponging miR−296‐5p
hsa_circ_0005556^b^	NBAS	chr2	Proliferation/invasion/chemotherapy resistance	CircRNA‐miRNA sponging sites: miR−548c−3p, miR‐ 587, miR−4739, miR−125a−3p, miR−297
circRNA_100269^b^	LPHN2	chr1	Proliferation	Targeting miR−630
hsa_circ_0000419^b^	RAB3IP	chr12	/	Harbor hsa‐miR−141‐5p and hsa‐miR−589‐3p by matching miRNAs seed sequences
circLMTK2^b^	LMTK2	chr7q21.3	Cell viability /mobility	Bax and cleaved Caspase 3 expression was enhanced significantly accompanying with suppressed Bcl−2
circ‐KIAA1244^b^	KIAA1244		Cellular components/molecular functions/ signaling pathways	/
Circular RNA_LARP4^b^	LARP4	chr12	Proliferation/invasion	Sponging miR−424‐5p and regulating LATS1 expression
Hsa_circ_0001614^b^	SENP6	chr6	Inflammatory cells	/
circSMARCA5^b^	SMARCA5	/	Proliferation/migration/ invasion	/
circPVRL3^b^	PVRL3	/	proliferation/ migration	Sponge to 9 miRNAs^d^ and be able to have a binding with AGO2, FUS, LIN28A, PTB, and EIF4A3
hsa_circ_0001017^b^	XPO1	chr2	/	/
hsa_circ_0061276^b^	NRIP1	/	/	/

^a^ Oncogene, Upregulated in gastric cancer tissue or plasma.

^b^ Suppressor, Downregulated in gastric cancer tissue or plasma.

^c^ hsa_circ_0103398, hsa_circ_0119099, hsa_circ_0121124, hsa_circ_0127859, and hsa_circ_0139915.

^d^ miR‐203, miR‐1272, miR‐1283, miR‐31, miR‐638, miR‐496, miR‐485‐3p, miR‐766, and miR‐876‐3p.

### 4.4 Effect of circRNA expression levels on disease‐free survival of GC

**FIGURE 4:**
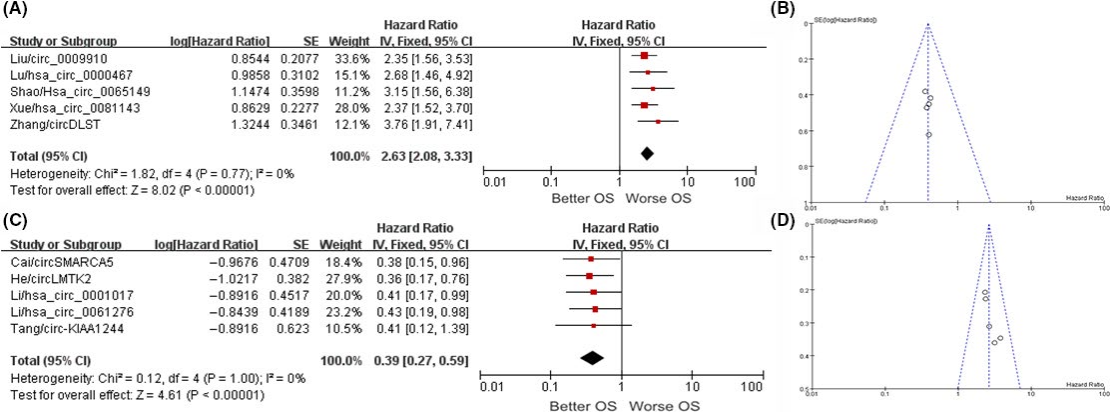
Forest and funnel plots for prognostic value of circRNAs in disease‐free survival (DFS) of gastric cancer patients. High‐expressing circRNAs indicate worse prognosis (A, B) or better prognosis (C, D) for gastric cancer patients

The included seven studies provided the HRs and 95% CIs of the disease‐free survival of GC patients, involving 1025 patients. We performed a quantitative analysis and found that there were four reports of high expression that were positively correlated with the disease‐free survival of patients. Quantitative analysis of total HR for the high‐expression group vs. the low‐expression group could be obtained (HR = 0.58; 95% CI, 0.43‐0.78; *p* = 0.0003). There was no heterogeneity, indicating that the research results were highly credible (Figure 4A); a funnel plot is shown in Figure 4B. At the same time, the high expression of circRNAs reported by another three reports was negatively correlated with the disease‐free survival of patients. The total HR for the high‐expression group vs. the low‐expression group could be obtained (HR = 1.66; 95% CI, 1.33‐2.07; *p* < 0.001).The results of the heterogeneity test also indicated that the research results were highly reliable (Figure 4C); a funnel plot is shown in Figure 4D. In summary, circRNAs can be used as biological markers that affect the quality of disease‐free survival and the prognosis of GC patients.

## 5 DISCUSSION

Because of chemotherapy resistance and tumor metastasis, the prognosis of GC is poor. Although new anticancer drugs continue to emerge, GC still poses a huge threat to human health. In recent years, increasing numbers of studies have focused on the clinical role of circRNAs. Many reports show that circRNAs can be used as molecular biomarkers for the prognosis of GC patients.^10^, ^12^, ^29^ Through a large data meta‐analysis, we did not focus on single circRNAs but provided evidence to elucidate the prognostic value of the abnormal expression of multiple circRNAs in GC patients. Furthermore, two groups of circRNAs with the largest HRs were selected to illustrate their prognostic value and provide ideas for future combined testing. The results of the meta‐analysis showed that the total HR value (95% CI) of OS and DFS was 1.83 (1.64‐2.03; *p* < 0.01) and 1.66 (1.33‐2.07; *p* < 0.01), respectively. The total HR (95% CI) of OS and DFS was 0.54 (0.45‐0.66; *p* < 0.01) and 0.58 (0.43‐0.78; *p* < 0.01), respectively. In addition, two panels of five circRNAs showed a greater prognostic value: 2.63 (2.08‐3.33; *p* < 0.01) (circ_0009910, hsa_circ_0000467, hsa_circ_0065149, hsa_circ_0081143, and circDLST); and 0.39 (0.27‐0.59; *p* < 0.01) (circSMARCA5, circLMTK2, hsa_circ_0001017, hsa_circ_0061276, and circ‐KIAA1244). These results indicated that abnormal expression of circRNAs can be used as tumor biomarkers for the prognosis of GC patients.

CircRNAs can promote or inhibit the process of tumor cell migration, invasion, proliferation, drug resistance, and epithelial‐mesenchymal transition (EMT). CircRNAs can function through different target genes, which is related to prognosis. For example, Li et al.^45^ showed that circ_104916 could inhibit the growth of GC. The expression level of circ_104916 in GC tissue and five GC cell lines was downregulated. Further experiments in vitro showed that circ_104916 could inhibit the proliferation, invasion, migration, and EMT of GC cells. The low expression of circ_104916 was related to poor prognosis. Wang et al.^17^ reported that circLMTK2, derived from exons 10 and 11 of *LMTK2* mRNA, has a negative effect on GC cells by inhibiting the function of *miR*‐*150*‐*5p* such as blocking DNA synthesis and reducing cell proliferation, migration, and invasion ability in GC cells. CircRNA‐circPDSS1 promotes the progression of GC by regulating *miR*‐*186*‐*5p* and *NEK2*.^21^ Wu et al.^37^ proved that circ‐DCAF6 is the upstream negative regulator of *miR*‐*1231* and *miR*‐*1256*, and its enhancement promotes cell proliferation and indicates a poor prognosis. CircRNA‐100269 can target *miR630* to inhibit the proliferation of tumor cells.^23^ CircLARP4, derived from exons 9 and exon 10 of *LARP4*, has high stability (half‐life >24 h), and it is also regarded as a potential biomarker. CircLARP4 can be regarded as the sponge of *miR*‐*424*‐*5p* to inhibit its effect of promoting cell proliferation and invasion. Therefore, the high expression of circLARP4 is related to better prognosis.^46^ CircRNAs can also induce apoptosis and inhibit cell proliferation, migration, and invasion in GC through the EMT signaling pathway.^47^ Xue et al.^41^ suggested that has_circ_0081143 might act as a ceRNA to inhibit *miR*‐*646*, increasing the expression of *CDK6* and promoting the resistance of GC to cisplatin. Another research group^48^ found that the expression of circAKT3 in GC tissues of cisplatin‐resistant patients was higher than that in cisplatin‐sensitive patients. Further mechanistic research showed that circAKT3 promoted DNA damage repair and inhibited cell apoptosis through sponging *miR198*. The process increased the expression of its downstream target *PIK3R1*, providing a new treatment prospect for cisplatin‐resistant patients. Shao et al.^49^ found that the expression of hsa_circ_0014717 was significantly downregulated in 77.2% of GC tissues. Its expression level in GC tissues was negatively correlated with tumor stage, distant metastasis, carcinoembryonic antigen, and CA19‐9. Therefore, the low expression of hsa_circ_0014717 was associated with poor prognosis. In addition, hsa_circ_0014717 was also detected in body fluids, indicating that hsa_circ_0014717 is a noninvasive biomarker. Another study^29^ reported that circPVT1 encoded by *PVT1* was upregulated in GC, which inhibited *miR*‐*125* and promoted cell proliferation. By analyzing the expression of circPVT1 in 187 patients with GC and their 100‐month follow‐up results, the researchers found that circPVT1 was an independent prognostic biomarker of OS and DFS in patients with GC. This suggests that circPVT1 not only plays a role in the treatment of GC, but also may be a prognostic marker for GC patients.

An important mechanism of circRNAs in cancer‐related signal transduction is circRNA‐miRNA‐protein. The PTEN/PI3K/AKT/mTOR signaling pathway is the most used research pathway between circRNAs and GC. CircPSMC3 indirectly upregulates *PTEN* through sponging miRNAs.^19^ Cirs7 targets *miR7* to inhibit the expression of *PTEN*, *AKT*, and *MTOR*. Therefore, Cirs7 can downregulate *PTEN* expression and upregulate *AKT* and *MTOR* expression.^8^ CircOSBPL10 has a carcinogenic role in GC by activating the Wnt/β‐catenin signaling pathway. Overexpression of circOSBPL10 will lead to upregulation of *WNT2* expression by sponging *mir*‐*136*‐*5p*. Wnt2 activates the Wnt/β‐catenin signaling pathway and promotes the development of GC.^50^ In addition, circHIPK3 and circHECTD1 also promote the development of GC through the Wnt/β‐catenin pathway.^15^, ^51^ The expression of circ‐ZFR in tumor tissue was significantly lower than in para‐carcinoma tissue, while the expression of circ‐ZFR in GC cell lines HGC‐27, AZ521, and AGS was significantly lower than that in the gastric epithelial cell line GES1. Circ‐ZFR causes cell cycle arrest and apoptosis of GC through sponging *miR*‐*107*/*miR*‐*130a*, which binds to the 3ʹ untranslated region (UTR) of *PTEN*.^52^ Many studies have shown that *PTEN* can be targeted and regulated by *miR*‐*107* and *miR*‐*130a*, affecting the activity of cancer cells.^53^, ^54^ These results suggest that the circ‐ZFR‐*miR*‐*107*/*miR*‐*130a*‐*PTEN* pathway has an important role in the development of GC. Although many signaling pathways related to GC have been described, the involvement of other signaling pathways such as the Notch pathway is still unclear.

There are still many deficiencies in our research. First, almost all tissue and plasma samples in our study were collected over a long period. Although the samples were preserved at a low temperature, there is still some influence on the degradation of circRNAs. Second, we cannot guarantee that all relevant studies are included in our analysis, and all the included studies with high prognostic value showed positive results. The publication bias reached a consensus. Thus, our results might overestimate the prognostic value of circRNAs among GC patients. Finally, we should perform more experiments to verify the reliability and economy of the analysis results in body fluid samples to judge whether circRNAs can be used as simple, fast, accurate, economic, and noninvasive tumor markers in clinical settings.

Evaluating the prognosis of patients and exploring the mechanism of circRNAs has a critical role in clinical decision‐making and in the development of novel targeted gene therapy. CircRNAs can perform physiological functions by acting as miRNA sponges and translation templates, regulating the expression of parental genes, and acting on RNA binding proteins. Despite many studies, we still know little about circRNAs, and they are far from being used in clinical practice. First, researchers mainly use tumor tissue or tumor cell samples to perform research on cancer‐related circRNAs, which should be extended to the detection of noninvasive samples (such as blood, urine, and saliva). Second, in the future, circRNAs may be considered as a potential therapeutic target. However, how to transport circRNAs to relevant sites to obtain long‐term efficacy and how to achieve nonimmune rejection are urgent problems. Finally, the goal of circRNA research is to apply them safely to the clinical treatment of human diseases, and thus, a large number of clinical studies are needed to complete this process.

## 6 CONCLUSION

By detecting the expression levels of a panel of circRNAs in tissues or body fluids, we can make appropriate clinical decisions based on different prognoses.

## CONFLICT OF INTEREST

The authors have no financial disclosures or conflicts of interest.

## AUTHOR CONTRIBUTIONS

H. Chen. and C. Liang had full access to all the data in the study and took responsibility for the integrity of the data and the accuracy of the data analysis. Study concept and design: X. Wang, Y. Liu; Manuscript drafting and reviewing: C. Ren; Acquisition, analysis, and interpretation of data: all authors; Statistical analysis: C. Liang; Administrative, technical, and material support: H. Chen. H. Chen and C. Liang contributed equally to this work.

## Data Availability

The data that support the findings of this study are openly available in [repository name e.g., “figshare”] at http://doi.org/[doi], reference number [reference number].
